# Advances in the Analysis of Veterinary Drug Residues in Food Matrices by Capillary Electrophoresis Techniques

**DOI:** 10.3390/molecules24244617

**Published:** 2019-12-17

**Authors:** Raffaella Colombo, Adele Papetti

**Affiliations:** Department of Drug Sciences, University of Pavia, V.le Taramelli 12, 27100 Pavia, Italy; raffaella.colombo@unipv.it

**Keywords:** food quality, food safety, veterinary drugs, animal food, food contaminants, capillary electrophoresis, residue analysis, pre-concentration techniques

## Abstract

In the last years, the European Commission has adopted restrictive directives on food quality and safety in order to protect animal and human health. Veterinary drugs represent an important risk and the need to have sensitive and fast analytical techniques to detect and quantify them has become mandatory. Over the years, the availability of different modes, interfaces, and formats has improved the versatility, sensitivity, and speed of capillary electrophoresis (CE) techniques. Thus, CE represents a powerful tool for the analysis of a large variety of food matrices and food-related molecules with important applications in food quality and safety. This review focuses the attention of CE applications over the last decade on the detection of different classes of drugs (used as additives in animal food or present as contaminants in food products) with a potential risk for animal and human health. In addition, considering that the different sample preparation procedures have strongly contributed to CE sensitivity and versatility, the most advanced sample pre-concentration techniques are discussed here.

## 1. Introduction

The addition of drugs (abuse or illegal use) to animal food to promote growth and protect animals can represent a potential risk of contamination of food matrices. Drugs, such as antibiotics, estrogens, non-steroidal anti-inflammatory drugs (NSAIDs), and β-agonists, which are usually used in feedstuffs, can contaminate different food products, mainly meat, milk, and dairy products, causing health problems and also serious diseases [1]. In particular, the abuse of antibiotics in food-producing animals, which contributes to the increase in risk of the transfer of antibiotic resistance from animals to humans, is a very important issue for human health. For this reason, the European Union (EU) and the Food and Drug Administration (FDA) established restrictive regulations for the control of pharmacologically active substance residues and fixed maximum residue limits (MRLs) in edible animal tissues to preserve foodstuff of animal origin and consumers [2,3,4].

The need of sensitive and rapid analytical techniques to detect and quantify pharmacologically active compounds, unauthorized drugs included, in animal food and foodstuff of animal origin has become mandatory for food security. Therefore, over the years, the necessity to develop and validate new analytical methods has increased [5]. The Commission Decision 2002/657/EC reported the technical guidelines and performance criteria for method validation for the control of the different residues [6]. In addition, there is a lack of regulation for veterinary drug residues (for example, for fluoroquinolones used as antimicrobials) in many foods, including baby foods [7].

EU guidelines suggest the use of the liquid chromatography (LC) technique; in particular, LC coupled with mass spectrometry (MS) was the most used approach to detect and analyze drug residues in complex matrices, such as milk and dietary products [8,9,10,11,12]. In addition, ion mobility spectrometry (IMS) coupled with MS represented a very promising powerful tool to detect analytes in traces [13].

Capillary electrophoresis (CE) with its well-known advantages, such as high efficiency, low consumption of sample and buffer, and rapidity, represents a potential alternative to LC methods in the analysis of drugs in different fields, including food analysis [14,15,16]. Another important advantage of CE rests in the versatility of applications thanks to the development of different CE separation modes. The simple addition of different molecules (surfactants, chiral selectors, polymers, particular electrolytes, and organic modifiers) to the buffer or the modification of the capillary inner wall with new packaging materials gave origin to different separation mechanisms and selectivity, increasing CE versatility and potential applications [16,17,18,19].

In particular, the use of electrospray ionization (ESI), matrix-assisted desorption/ionization (MALDI), and inductively coupled plasma (ICP) as CE-MS interfaces improved food analysis by increasing CE sensitivity [20,21]. In fact, CE-MS represents the ideal technique to detect analytes in traces with important implications in food contaminants and residue analysis [15,16].

In addition, advances in electrochemical detectors, such as CE-contactless coupled detection (CE-CCD) and CE-capacitively coupled contactless conductivity detection (CE-C^4^D), offered very sensitive methods [22,23]. Furthermore, the development of miniaturized CE systems (microchip-CE devices) allowed the monitoring of food analytes with rapidity and sensitivity, and their use was particularly important in the detection of frauds or contaminations [24,25,26].

Due to the complexity of food matrices, which are mainly rich in lipids, carbohydrates, and proteins, a pre-concentration step was necessary to detect drug residues in trace amounts. This became mandatory because of the intrinsic poor sensitivity of CE [27]. Solid phase extraction (SPE) and miniaturized SPE are the most used procedures, not only for the pre-concentration step, but also for the sample clean-up. New SPE sorbents with high adsorption capacity and high resistance were studied, giving origin to selective materials for some drugs and also generating advanced high-throughput procedures able to extract different drug classes [28,29]. In addition, advances in on-chip SPE-CE procedures also allowed low-abundance analytes with high sensitivity to be analyzed [30]. Recently, the combination of traditional liquid-liquid extraction (LLE) and SPE procedures or advanced liquid extraction techniques, such as dispersive liquid-liquid microextraction (DLLME), was successfully applied to detect analytes in trace, increasing CE sensitivity [31,32,33].

Finally, the development of on-line procedures in which pre-concentration techniques were integrated with the CE instrumentation had many advantages, such as minimal sample loss, low cost, and rapidity [34].

In this review, we focused the attention on the potential of CE in veterinary drug residue analysis, considering the versatility of different CE-modes (mainly capillary zone electrophoresis, CZE; capillary electrochromatography, CEC; micellar electrokinetic chromatography, MEKC; nonaqueous capillary electrophoresis, NACE). CE methods and advanced sample preparation procedures combined with CE techniques in the last decade were also discussed. The main CE-modes were summarized, subdividing drugs into antibiotics (classified according to different molecular structures) and other drugs (estrogens, non-steroidal anti-inflammatory drugs, NSAIDs, and β-agonists).

## 2. Antibiotics

### 2.1. Nitroimidazoles

5-Nitroimidazoles (5-NDZs) are mainly active against Gram-negative and Gram-positive anaerobic bacteria. Some benzimidazoles (BZs) are also used as additives in stored fruit and vegetables, because of their fungicidal properties [35]. Regarding the veterinary use, these substances are prohibited [2,36], as they could be a potential risk for human health because of their genotoxicity and mutagenicity [37]. Therefore, no MRLs were established.

A rapid CEC-UV method for the analysis of 5-NDZ residues in bovine milk samples was set-up by Hernández-Mesa et al. The characteristic speed and high efficiency of CEC, which combines mobility and partition principles, are well-known to be affected by the complexity in fritting fabrication, which gives origin to pressure drops and bubble formation with loss of efficiency and reproducibility. In this work, the authors overcame this problem by proposing a simple capillary packing procedure and optimizing a sintering process (parameters: Time and temperature), thus obtaining a reproducible frit fabrication. A proper set-up of buffer composition and concentration, capillary length, and voltage allowed a reproducible analysis of eight 5-NDZs in milk samples in 15 min. In addition, a sample pretreatment using LLE and SPE methods highly increased the method sensitivity (LOQ range for all the 5-NDZs analyzed was 19–96 μg/L) [38]. This method, combining the advantages of CE and HPLC, was more selective and rapid than the simple HPLC [39] or CZE [40] methods.

The same technique (CEC-UV), but coupled with DLLME as a pre-concentration step, was used to analyze BZs in environmental and farm water [41]. DLLME, which belongs to liquid-phase microextraction (LPME) techniques, is based on the formation of a dispersion, created by adding an organic solvent mixture to an aqueous sample. It represented an ideal procedure to efficiently extract environmental pollutants and an interesting greener approach in their analysis [42].

This procedure was also used by Hernández-Mesa et al. to concentrate different 5-NDZ compounds in river water samples before the analysis with a cation-selective exhaustive injection and sweep (CSEI-sweep)-MEKC-UV method. DLLME and CSEI-sweep approaches combine an electrokinetic injection of charged cations and a sweeping in which the formation of micelles is promoted to focus the analyte. Thus, the MEKC method was more rapid, allowing the separation of six BZs in about 10 min [43]. The DLLME procedure coupled with MS detection could also improve the sensitivity of CZE. An example was the study of Tejada-Casado et al., who set-up a CZE-MS/MS method able to rapidly (about 30 min) detect and quantify twelve BZs in meat samples, as a valid alternative to HPLC methods [44].

Finally, SPE combined with CSEI-sweep-MEKC-UV allowed six nitroimidazole residues in egg samples to be quantified with LOQ values in the range of a few ng/g [45].

### 2.2. Fluoroquinolones

Quinolones represent a class of drugs commonly used in veterinary fields, whose MRLs have been established in the range level of 100–500 μg/kg by the EU Council Regulation and FDA [2,3]. Fluoroquinolones (FQs) are synthetic quinolones, related to nalidixic acid, which act against Gram-negative and Gram-positive bacteria by inhibiting their DNA synthesis. FQs are commonly used for livestock growth and aquaculture and are toxic to human health because they can exhibit a direct toxicity, responsible for muscular and neuronal dysfunctions, or cause antibiotic resistance or allergies [46,47,48].

CE techniques also represented interesting platforms in the analysis of the third and fourth generation of FQs in different food matrices (water, milk, and animal muscle), but appropriate sample pre-treatments and detection systems (LIF and MS) became mandatory to obtain a high level of sensitivity [49]. To extract milk FQs, LLE and SPE were the most used procedures, but in the literature, examples of protein precipitation (PPT) followed by SPE are also present as a good alternative [50]. In particular, molecularly imprinted polymers (MIPs), which are selective and stable sorbent materials for SPE (MISPE technique) [51], allowed promising CZE-LIF or CZE-MS methods to be obtained in the animal foodstuff analyses. MIPs can be used both before CZE analysis and as an in-line-MISPE strategy, thus obtaining sensitive and selective methods for the analysis of different complex matrices (for example, pig kidney and bovine milk) [52,53]. In fact, by using advanced MIP technologies, the low CZE-UV sensitivity can also be overcome. Magnetic molecular imprinted polymers (MMMIPs) were prepared by combining ferroferric-oxide nanoparticles, and MIPs and had a rapid and efficient binding capacity. These materials allowed a rapid and selective CZE-UV method to be obtained to separate fleroxacin, gatifloxacin, lomefloxacin (LOM), and norfloxacin in bovine milk samples [29]. The method sensitivity (LOD range: 12.9–18.8 μg/L) was slightly lower than, but comparable to, that of Springer et al. (7.5–11.6 μg/L), who separated ciprofloxacin (CIP), norfloxacin, and ofloxacin (OFL) in milk samples using a miniaturized SPE (made of carbon nanotubes as sorbents with high adsorption capacity and stability) to prepare samples [28].

Lara et al. set-up a CZE-MS/MS method for the simultaneous quantification (ng/kg level) of eight FQs, i.e., danofloxacin, sarafloxacin, CIP, marbofloxacin, enrofloxacin (ENRO), difloxacin, oxolinic acid, and flumequine, in chicken muscle samples. Two different sample preparation approaches were set-up; the first one consisted of pressurized liquid extraction (PLE), which was performed in an accelerated solvent extraction, followed by centrifugation, percolation, concentration, reconstitution, and filtration steps prior to the injection into the CE instrument; the second one was an in-line SPE, using a mixed-mode sorbent (RP and ion-exchange sorbents), in which different parameters, such as sample pH, volume, elution plug composition, and injection time, were optimized. The combination of in-line SPE-CE-MS/MS with PLE improved the selectivity and sensitivity, and this was particularly useful in multiresidue analysis [54].

Field-amplified sample stacking (FASS) with sweeping represented another on-line pre-concentration procedure used in combination with CE to rapidly quantify ENRO and CIP (LODs ng/mL) in milk and animal tissues. The FASS procedure consists of an electrokinetic injection of sample in a run buffer with high conductivity, creating an interface in which the difference in the electric field between the sample matrix and run buffer promotes sample stacking. The use of gamma-cyclodextrin in the sample matrix (sweeping procedure) could increase the FASS sensitivity, because it gave origin to micelles as in the MEKC technique, in which cyclodextrins are added to the background electrolyte (BGE) [55].

A different on-line preconcentration procedure, named field-enhanced sample injection (FESI), could increase CZE-UV sensitivity. FESI is useful for samples with low conductivity and consists of a careful optimization of the injection (pressure and time) of a water plug into the capillary in order to increase the capillary electric field by creating a sample stacking effect. The use of FESI in addition to a bubble cell capillary (with a longer window pathway than the traditional capillary) increased the method sensitivity for five different FQs (ENRO, CIP, LOM, fleroxacin, and OFL) in bovine milk samples [56].

Another CE mode, i.e., nonaqueous capillary electrophoresis (NACE)-UV, combined with a DLLME approach allowed the set-up of a selective method for the separation and quantification of eight FQs (omefloxacin, levofloxacin, marbofloxacin, CIP, sarafloxacin, ENRO, danofloxacin, and difloxacin) in water samples [57]. In NACE, BGE is added with organic solvents, mainly methanol or acetonitrile, and this promotes the separation of low-water-soluble molecules. In fact, even if the use of organic solvents could induce changes in the pKa values and mobility, the advantage consisting of an increased selectivity becomes fundamental [16]. The same CE-mode coupled with an in-line single-drop liquid-liquid-liquid microextraction (SD-LLLME) was used to separate CIP and ENRO in surface and groundwater samples. This setup allowed a reduction in the analysis time (as no sample pre-treatment was necessary) and in the volume of extraction solvent, as a buffer was used as a pH donor, a drop of NaOH as a high pH acceptor, and an organic solvent as a medium in which the analytes diffused [58].

### 2.3. Tetracyclines

Tetracyclines are widely used as economic broad-spectrum antibiotics against both Gram-positive and Gram-negative bacteria. MRLs were established by the Commission Regulation (EU) and ranged from 100 to 600 μg/kg, depending on animal tissues or the food sample [2]. In the literature, many works aimed to analyze and quantify these antibiotics. A simple SPE procedure with a NACE-LIF method allowed a very sensitive separation of chlortetracycline (CTC), tetracycline (TC), oxytetracycline (OTC), and doxycycline (DC) in feeds and milk with pg/mL LOD values [59]. In the same year, Deng et al. proposed a simple CZE-enhanced chemiluminescence (ECL) method to monitor TC residues over time in crucian carp muscle of fish samples, with a sensitive detection under MRL values [60].

Recently, a field-amplified sample injection (FASI) procedure in CZE-UV was set-up for the detection of four tetracyclines in pig farms’ wastewaters with results comparable to those obtained by HPLC-UV methods [61]. FASI pre-treatment is particularly suitable for large amounts of water samples and is based on a difference in electrical conductivity between the sample and the background electrolyte, which causes a stacking effect responsible for the increase in peak efficiency and method sensitivity.

**Figure 1:**
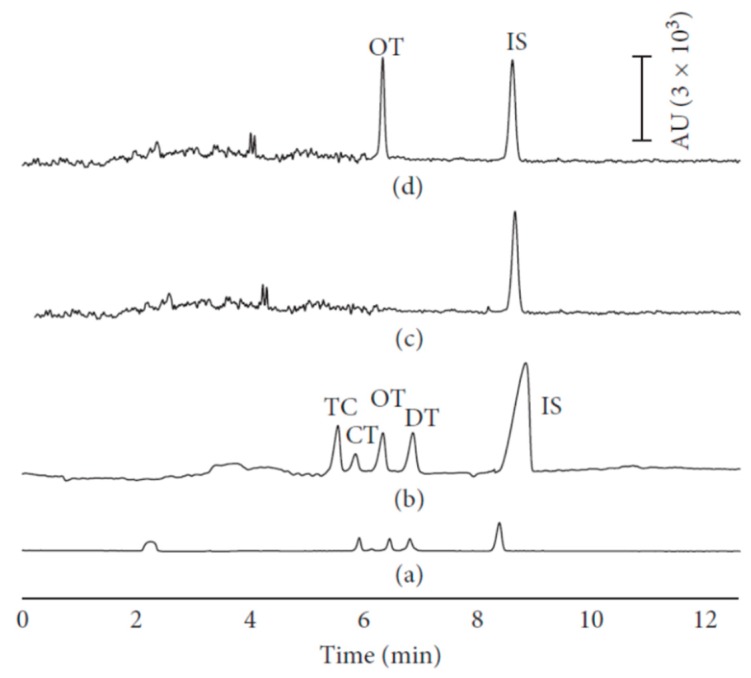
Solid phase extraction (SPE)-large-volume sample stacking (LVSS)-capillary electrophoresis (CE) method applied to milk sample for the detection of tetracyclines (TCs). Electropherograms of (**a**) standard TC sample (10 mg/L) analyzed by CE, (**b**) standard TC sample (1 mg/L) analyzed by LVSS-CE, (**c**) blank milk sample, and (**d**) real milk sample analyzed by SPE-LVSS-CE method [62].

The combination of two pre-concentration procedures could be useful to determine drug residues, even when present at a concentration below MRL limits. Islas et al. recently set-up a SPE step followed by a large-volume sample stacking (LVSS) approach before a CZE-UV analysis of TC. LVSS consisted of a stacking procedure with an on-line series of polarity switches (PS) and enabled an improvement in sensitivity and reproducibility, particularly useful for low-concentration analytes in complex matrices, such as milk samples (Figure 1) [62]. The same pre-concentration approach (SPE-LVSS-PS) was previously used to determine five TC residues (metacycline-MTC, OTC, TC, CTC, and DC) in water samples, reaching a high sensitivity (ng/L) in a short time (10 min) [63]. The LVSS approach alone, after a careful optimization of stacking conditions (sample zone length and stacking time), allowed a CZE-UV method to be obtained with a sensitivity around 10 ppb, a sensitivity level very similar to that obtained by using ED or LIF detectors. This method was used to detect TC, CTC, OTC, and DC in tap water samples [64].

Another promising extraction approach consisted of matrix solid-phase dispersion (MSPD), which required the addition of sorbents to the sample with a consequent elution step before the analysis. Mu et al. set-up an economic MSPD-CZE-UV method to rapidly (about 6 min) separate TC, OTC, and DC in milk samples [65]. In order to improve CE sensitivity, a functionalized β-cyclodextrin-ionic liquid was added in-line; unlike the conventional β-cyclodextrin, it acted as an additive able to form a complex with TCs and as a capillary coating agent. The method proposed by Zhou et al. was developed to separate four TCs in about 30 min by using CE with amperometric detection (AD) [66].

Tetracyclines can also be present in honey, representing a serious problem, as the EU Commission did not admit to the use of antibiotics in honey and did not establish MRLs for bee products [2]. Casado-Terrones et al. set-up an SPE procedure followed by a CZE-UV method to simultaneously and rapidly (16 min) determine eight tetracyclines in honey with LOD values of 23.9–49.3 μg/kg [67].

### 2.4. Sulfonamides

These molecules are widely used for the treatment of bacterial and protozoan diseases (i.e., malaria) and as growth-promoters in farm animals. The EU fixed MRLs to 100 μg/kg in different animal tissues and milk [2]. Sulfonamides could be dangerous for human health. For example, studies on sulfamethazine carcinogenesis are very controversial, but nowadays, it is not yet classified as carcinogenic by the U.S. National Toxicology Program 14th Report on Carcinogens (2016).

For the antimalarials sulfadoxine and quinine, the old literature proposed CZE-UV as the main CE-mode in meat and water analysis with sensitivity values of μg/L and mg/L for sulfadoxine and quinine, respectively [68]. More recently, Mikus et al. set-up a capillary isotachophoresis (CITP) coupled on-line with the CZE method to detect quinine in commercial beverages. This approach could be potentially used to detect drug residues in food, as CITP combined on-line with CZE-UV significantly increased the CZE sensitivity. In fact, in CITP, leading and terminating electrolytes are used to create separated zones in which the ions migrate at the same velocity, giving origin to an on-line stacking effect [69]. This combination allowed the direct analysis of samples with a lower LOD than CZE-UV methods and with sensitivity values comparable to those obtained with HPLC-UV methods [70].

**Figure 2:**
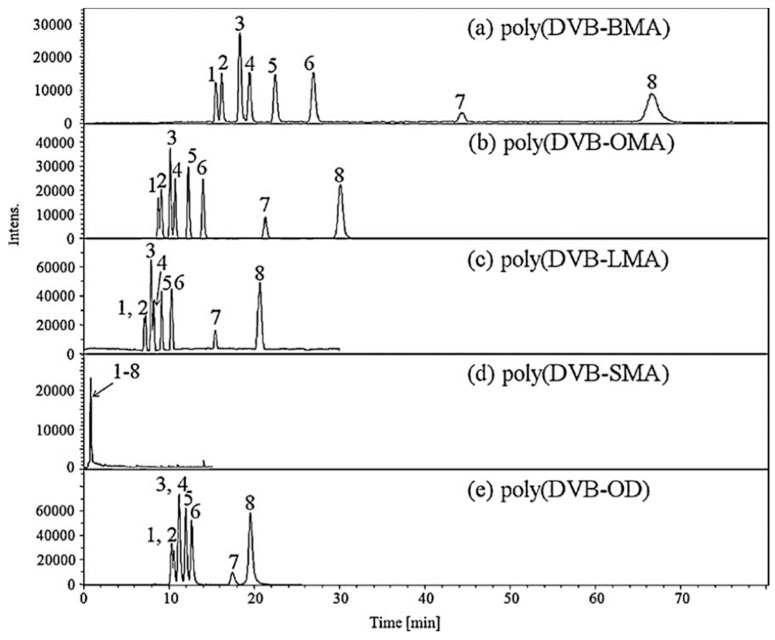
Capillary electrochromatography (CEC)-electrospray ionization (ESI)-mass spectrometry (MS) electropherograms obtained by using different monolithic stationary phases in the analysis of standard sulfonamides (**a**–**e**). The use of poly(DVB-OMA) capillary (**b**) allowed the best compromise to be obtained between resolution, efficiency, and analysis time [71].

Nine sulfonamides in meat samples were efficiently separated by CEC-ESI-MS. The use of a poly(divinylbenzene-octyl methacrylate) (poly-DVB-OMA) monolithic stationary phase and an on-line concentration (obtained increasing sample injection time) provided a sensitive method for detecting trace residues that needed only a simple sample pretreatment (i.e., SPE) (Figure 2) [71].

Wang et al. set-up a microfluidic CE system with LIF detection, which was able to separate four sulfonamides (sulfamethazine, sulfamethoxazole, sulfaquinoxaline, and sulphanilamide) in milk and chicken muscle extracts in 1 min with LOD values of a few μg/L. The short time required for the analysis and the fact that the plastic chips proposed were cheap made this method very useful for a rapid screening of sulfonamide residues in food samples [72].

Recently, Dai et al. proposed a CZE method with on-line ECL detection to quantify sulfadimidine, sulfadiazine, and sulfathiazole in pork and chicken meat samples. Chemiluminescence (CL) emissions were generated by the oxidation of luminol in the Ag(III)-luminol system, and sulfonamides exhibited an inhibitory effect on CL signals. The careful optimization of different parameters (buffer type and pH, voltage, and injection time) allowed a promising selective and sensitive method to be obtained for the analysis of veterinary drug residuals in animal-derived food [73].

### 2.5. Aminoglycosides

These antibiotics (AGs) are widely used in veterinary medicine for bacterial and protozoan infections. In addition, AGs are frequent honey contaminants from non-EU countries, which is a big issue. As mentioned above, the use of antibiotics in beekeeping is not authorized in the EU [2] and, therefore, there are no European Community regulations about MRLs for these drugs in this product. To detect these compounds in other complex matrices, i.e., biological fluids and pharmaceutical samples, CE with LIF, C^4^D, or indirect detections, are requested [74]. Recently, Moreno-Gonzalez et al. detected nine AGs (three gentamicins, neomycin, apramycin, paromomycin, dihydrostreptomycin, spectinomycin, and streptomycin) in different types of honey using MISPE and FASS procedures as purification and pre-concentration steps, and a CZE-MS/MS analysis method [75]. MISPE also represented an interesting tool for the extraction and analysis of AGs in different animal-derived food (meat) [76].

In 2019, a microchip-CE-C^4^D method was developed using standard solution and was proposed as a good alternative to separate in a very short time (less than 1 min) AG sulfates (gentamicin sulfate, kanamycin sulfate, and streptomycin sulfate) in foodstuff with LOD values of 1–3 μg/mL [77].

### 2.6. Macrolides

Macrolides are bacteriostatic antibiotics with a broad spectrum of activity against Gram-positive and Gram-negative, particularly used in the treatment of respiratory diseases in bovines and pigs, but generally also added to animal feed. Like aminoglycosides, they are listed in Group B1-antibacterial substances under the Council Directive 96/23/EC [78] and possess the same mechanism of action consisting of the inhibition of bacterial protein synthesis, but they bind the 50S instead of 30S ribosomal unit. Their presence in food could cause problems to human health, in particular, to intestinal bacterial flora, and, in the last years, their use for long periods contributed to cause the development of antibiotic resistance [79,80]. An EU directive established MRL values in the range 40–200 μg/kg for erythromycin, tylosin (TYL), tilmicosin (TIL), and spiramycin [2,80].

For the evaluation of macrolides in food, HPLC-UV or HPLC-ESI/MS were the most frequently used methods [81,82]. However, off-line or on-line pre-concentration steps were the ideal approach to overcome the derivatization step required to obtain a high sensitivity by CE. Among the on-line pre-concentration techniques, FASS represented a promising solution, as demonstrated in the analysis of TIL, erythromycin, clarithromycin, roxithromycin, and tylosin residues in milk samples by a FASS-MEKC-UV method (LOD range of 0.002–0.004 mg/kg) [83]. CZE-DAD was recently proposed to analyze macrolides (TYL and TIL) in chicken fat samples, reaching LOQ values of a few μg/kg thanks to a preconcentration step that consisted of an evolution of the DLLME procedure: In fact, the extraction and the preconcentration occurred in an ionic liquid by using ultrasound instead of dispersive solvents (this procedure is named reverse ultrasound-assisted emulsification-microextraction, R-USAEME). Organic salts, which are more stable and less toxic than the organic solvents commonly used in DLLME, were used to prepare the ionic solution [84].

### 2.7. β-Lactam Antibiotics

Penicillins and cephalosporines are classified as β-lactam antibiotics. They have the same action mechanism, but cephalosporines have a more extended spectrum. In particular, in veterinary medicine, ceftiofur and cefquinome are specifically used to treat respiratory diseases and exudative epidermitis, and meningitis, respectively. Cefquinome had MRL values more restrictive than those of ceftiofur (50–200 μg/kg vs. 100–6000 μg/kg) [2], but the use of ceftiofur must be carefully evaluated as its use could develop E. coli resistance [85,86].

CZE and MEKC modes were the best choice for the analysis of penicillins and cephalosporins in complex matrices [87,88]. For example, penicillin acid and penicillin G could be easily detected in milk by CZE-UV with a simple sample pre-treatment, which consisted of the deproteinization, extraction, and precipitation of milk protein with acetonitrile [89]. To achieve the best resolution, an on-line sample concentration step coupled with a CEC-MS method with a polymeric monolithic column was proposed by Liu et al. The injection in CE consisted of an anion selective injection (ASEI) performed by solubilizing the analytes in buffers with different pH values and promoting a stacking effect. This method was applied to analyze milk samples, obtaining a high sensitivity [90].

Regarding cephalosporins, Hancu et al. set-up a simple and rapid CZE-UV method, simply optimizing BGE composition and pH. The method was able to separate seven cephalosporins in 6 min and it was proposed for the analysis of pharmaceutical products and different complex matrices, obtaining very low LOD values; therefore, it could be useful for residue analysis [88].

### 2.8. Simultaneous Analysis of Different Antibiotics

The separation and quantification of different drugs (macrolides and tetracycline antibiotics) in feedstuffs could be simultaneously carried out by CZE-UV, as demonstrated for TIL, TYL, TC, OTC, and DC with LOD values of 0.5–1 mg/kg [91]. The method sensitivity was good considering that OTC and TYL were added in feeds for growth promotion in a range from a few to 50 mg/kg, and that TIL was also often added as an antimicrobial and respiratory diseases agent at a concentration of a few hundreds of mg/kg [92]. The separation was performed in 15 min and, thus, this CE-method could be considered useful for rapid routine analysis.

In addition, β-lactams, tetracyclines, quinolones, amphenicols, and sulphonamides were simultaneously and rapidly (8 min) separated by CZE-UV in bovine raw milk. The combination of LLE and SPE extraction procedures before CE-analysis allowed LOQ values lower than MRLs to be obtained [93].

Another example of a simultaneous separation of different antibiotics (fluoroquinolones, tetracyclines, and β-lactams) in milk was proposed by Long et al. The use of ECL detector allowed trace to be detected, reaching LOD values of cents and thousands of μg/mL [23].

If carefully optimized, CE methods could be very promising in routine screening as good alternatives to LC-MS methods. For example, Kowalski et al. set-up a SPE procedure combined with a MEKC-UV method able to selectively and successfully separate sulfonamides (sulfamethazine, sulfamerazine, sulfathiazole, sulfachloropyridazine, sulfamethoxazole, sulfacarbamide, and sulfaguanidine) and amphenicol-type antibiotics (chloramphenicol, thiamphenicol, and florfenicol) in commercial poultry samples (muscle, liver, and skin with fat) [94].

More recently, the use of FASS combined with a micelle to the solvent stacking (MSS) approach allowed the set-up of a very sensitive CZE-UV method to detect sulfamethoxazole and trimethoprim (an antibacterial agent frequently used in combination with sulfamethoxazole for respiratory and urinary infections) in dairy products, chicken eggs, and honey. MSS consisted of an injection of a micellar solution plug prior to FASS, in order to obtain a focused sample zone with an increase in CE sensitivity [95].

In 2019, a microchip-CE with an LED-induced fluorescence detector was used as a promising platform to simultaneously analyze antibiotics in food. The chip was tested for chloramphenicol (CAP) and kanamycin (Kana) quantification in milk and fish samples, obtaining rapid analysis (3 min) with LODs of pg/mL. Unlike other microchip-CE methods, which had the disadvantages of complexity and low versatility, this platform used a simple strategy, named stir-bar assisted DNA multi-arm junctions recycling, which exploited the capacity of a gold bar with the DNA probe to capture antibiotics, allowing a multiplexed detection and increasing the method sensitivity without matrix interference [26].

To analyze fluoroquinolones and sulfonamides in environmental water, He et al. set-up an on-line preconcentration procedure with a pressure-assisted electrokinetic injection (PAEKI) [96]. PAEKI resolves FASI limits in the analysis of anionic molecules, which can be depleted when the voltage is applied in reverse mode. PAEKI parameters (pressure and voltage) were optimized, obtaining LOD values of a few μg/L and improving the results obtained with hydrodynamic or electrokinetic injections [96].

## 3. Other Drugs

### 3.1. Estrogens

Estrogens are widely used in intensive farming worldwide. In EU and United States livestock, the discharge of estrogens is about 83,000 kg/year, representing a very dangerous environmental pollutant. Their low water solubility and the fact that they can easily be degraded and transformed contribute to make them important water contaminants [97].

Wu et al. proposed the use of SPE and pressurized CEC (*p*-CEC)-AD to separate five estrogens (bisphenol-A, 4-tert-octylphenol, 4-*n*-nonylphenol, 2,4-dichlorophenol, and pentachlorophenol) in chicken eggs and milk powder samples. *p*-CEC is an advancement of CEC, in which the formation of typical air bubbles of CEC is prevented by setting a micro-HPLC pump at the inlet of the CE capillary. The set-up method was selective and exhibited a 100- to 500-fold higher sensitivity than CE-UV methods, with values comparable to GC-MS methods [98].

The first DLLME approach combined with MEKC-ESI/MS to extract and analyze estrogens (estriol, 17α-estradiol, 17β-estradiol, estrone, 17β-ethinylestradiol, and their main metabolites) in different milk samples and milk derivatives was set-up by D’Orazio et al. The method was simple, less expensive, and allowed a μg/L LOD level to be obtained [32]. DLLME and MEKC-UV were also used in the analysis of hexestrol, bisphenol A, diethylstilbestrol, and dienestrol, which are phenolic environmental estrogens (PEEs) frequently present in water as contaminants. In this case, after an optimization of DLLME procedure (type and volume of extraction solvent; volume of dispersive solvent; extraction time; salt concentration), LOD values (0.3–0.6 μg/L) within the requirements of trace analysis in environmental water were obtained [33].

### 3.2. Non-Steroidal Anti-Inflammatory Drugs (NSAIDs)

NSAIDs are added to animal feed mainly to treat respiratory diseases and allergies, generally in association with antibiotics. In addition, they could also improve animal meat quality by reducing fats [99]. They were classified as group B substances and, for many of them, MRLs were established by the European Council in relation to different animals and food matrices [2].

Diclofenac was banned in dairy animals, and other commonly used molecules (ketoprofen, salicylic acid and salicylates, acetylsalicylic acid, and acetylsalicylates) were approved only for non-dairy animals or for animals not involved in egg production [99]. For ibuprofen and flurbiprofen, no MRL value was established, and this could represent a serious problem as NSAIDs cause important side effects (from gastrointestinal problems to cancer) in humans [100].

Regarding the analysis of NSAIDs in animal feed, LC-MS was the most widely used approach, reaching LOQ values around ng/mL [10,101]. Nevertheless, off-line and on-line stacking procedures could lead to promising sensitive CE methods. Alshana et al. set-up a rapid DLLME-FASS-CZE method to detect five main NSAIDs (etodolac, naproxen, ketoprofen, flurbiprofen, and diclofenac) and their derivatives in bovine milk with results similar to those obtained using conventional SPE-LC techniques. In fact, DLLME and FASS combined extraction and stacking procedures are able to develop a sensitive method with a 1000-fold decrease in LOQ values (μg/kg), in comparison to conventional CZE techniques [99]. Among NSAIDs, naproxen, ketoprofen, and clofibric acid are widely used in veterinary medicine and represent contaminants of emerging concern (CECs) for the aquatic environment. SPME is a rapid off-line pre-concentration step ideal for the analysis of highly polar compounds, as NSAIDs, in water samples, with high sensitivity. This was demonstrated by Espina-Benitez who set-up a rapid and economic synthesis of new coated fibers for SPME, developing a sensitive CZE-UV method [102].

### 3.3. β-Agonists

β-agonists are widely used not only as bronchodilators, but also as muscle growth promoters to increase bovine, lamb, and pork meat production, mainly in Asian countries. They can be toxic for human health, particularly for their effect on the cardiovascular system, and, for this reason, the EU set a very low MRL for clenbuterol (0.05–0.5 μg/kg) in meat and milk samples [2].

NACE coupled with MS (NACE-MS) allowed trace amounts of β-agonists (clenbuterol, salbutamol and terbutaline, TER) to be detected. Anurukvorakun et al. developed a NACE-MS set-up, obtaining results comparable to those obtained by an HPLC-MS/MS method. In fact, the sensitivity is very high thanks to the combination of an SPE using mixed-mode reversed phase/cation exchange cartridges and hydrodynamic and electrokinetic injections in the CE-system [103].

For the quantification of ractopamine and dehydroxyractopamine in porcine meat, Wang et al. proposed an interesting MEKC-UV method with results totally in agreement with those obtained by MS techniques. The method was an on-line stacking method, including exhaustive CSEI and sweeping with a 900 fold higher sensitivity (ng/g level) in comparison to CZE-UV. The capillary was filled with a long plug of a higher conductivity buffer and an electrokinetic injection of the sample was then performed; at the end, the compounds were separated by a sweeping buffer with SDS, whose electrostatic forces contributed to the mobility and resolution of the two analytes [104]. Similarly, FASI-sweep-MEKC improved the MEKC sensitivity by 400–2000 times (ng/mL), allowing the analysis of eight β-agonists (R-albuterol hydrochloride, cimaterol, clenbuterolhydrochloride, colterol, TER, tulobuterol, ractopamine hydrochloride, and zilpaterol) in animal feed samples. A very efficient method was proposed by combining the sensitivity derived from the stacking effect of the FASI-sweeping procedure with the particular selectivity of a dialkyl-chain anionic surfactant in place of SDS [105].

**Figure 3:**
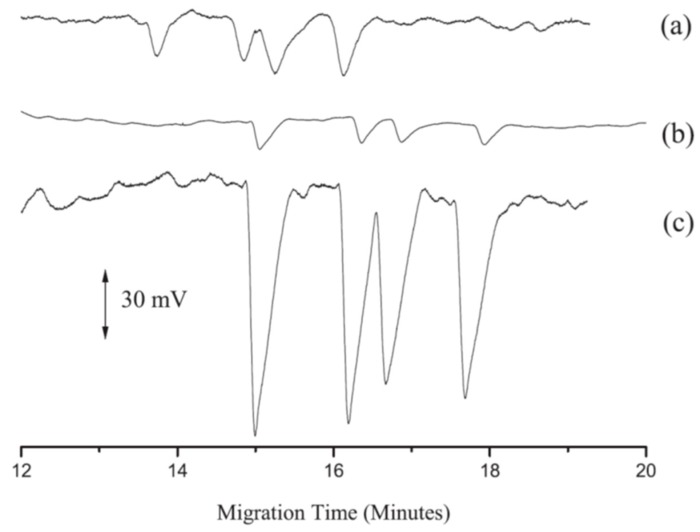
CE-capacitively coupled contactless conductivity detection (CE-C^4^D) electropherograms of four β2-agonists in pig feed. (**a**) Samples (10 mg/L in background electrolyte (BGE)) without pre-concentration step; (**b**) samples (0.1 mg/L in methanol) with field-enhanced sample injection (FESI) preconcentration step; (**c**) samples (5 mg/L in methanol) with FESI preconcentration step [106].

The combination of FESI and CE-C^4^D represented another important alternative in the analysis of these drugs in animal feed. In 2014, Gao et al. proposed the first method able to detect TER, procaterol, formoterol, and bambuterol in pig feed. FESI as a pre-concentration step allowed an improvement in LOD values (0.02 mg/L) to be obtained in comparison to UV detection (Figure 3) [106].

## 4. Conclusions

In the last years, the use and abuse of veterinary drugs has represented a very important issue for animal and human health. Consequently, the need of sensitive and rapid analytical techniques for the analysis of veterinary drug residues in food products has become a challenge. This review highlighted the potential role of CE in this topic, as summarized in Table 1. In fact, it is evident that the recent progress in sample pre-concentration methodologies coupled to CE allowed the sensitivity limits of this technique to be overcome and to improve its versatility in the analysis of different types of molecules, in complex food matrices, too.

## Figures and Tables

**Table 1:** CE-modes in the analysis of the mainly used veterinary drugs in different food matrices. Method sensitivities in terms of LOD or LOQ values are reported.

CE-Technique	Food Matrix	Sensitivity	Ref.
	**Nitroimidazoles**		
LLE-SPE-CEC-UV	bovine milk	LOQ: 19–96 (μg/L)	[38]
DLLME-CEC-UV	water	LOQ: 5.7–9.3 (μg/L)	[41]
DLLME-CZE-MS/MS	poultry and porcine meat	LOQ: 4–16 (μg/kg)	[44]
DLLME-CSEI-sweep-MEKC-UV	water	LOQ: 2.05–8.14 (ng/mL)	[43]
SPE-CSEI-sweep-MEKC-UV	egg	LOQ: 6.99–16.8 (ng/g)	[45]
	**Fluoroquinolones**		
SPE-CZE-UV	bovine milk	LOD: 7.5–11.6 (μg/L)	[28]
MMMIPs-CZE-UV	bovine milk	LOD: 12.9–18.8 (μg/L)	[29]
PPT/SPE-CZE-UV	bovine milk	LOQ: 0.06–0.1 (mg/kg)	[50]
MISPE-CZE-LIF	bovine milk, pig kidney	LOQ: 0.55–35 (μg/kg)	[52]
MISPE-CZE-MS/MS	bovine milk	LOQ: 3.2–4.7 (μg/kg)	[53]
PLE-SPE-CZE-MS/MS	meat	LOQ: 130–470 (ng/kg)	[54]
FASS-sweep-CZE-UV	milk, meat	LOD: 5.70, 7.39 (ng/mL)	[55]
FESI-CZE-UV and CZE-MS	bovine milk	LOQ: 2.3–8.3 (μg/kg)	[56]
DLLME-NACE-UV	water	LOQ: 5.43–461 (μg/L)	[57]
SD-LLLME-NACE-UV	water	LOD: 10.1, 55.3 (μg/L)	[58]
		**Tetracyclines**	
CZE-ECL	fish	LOD: 1.8 ng/mL	[60]
FASI-CZE-UV	water	LOQ: 23–59 μg/L	[61]
SPE-LVSS-PS-CZE-UV	milk	LOD: 18.60–23.83 (μg/L)	[62]
MSPD-CZE-UV	milk	LOD: 0.0745–0.0808 (μg/mL)	[65]
SPE-LVSS-PS-CZE-UV	water	LOQ: 67–167 (ng/L)	[63]
LVSS-CZE-UV	water	LOD: 8.1–14.5 (μg/L)	[64]
CZE-AD	water	LOD: 0.33–0.67 (μM)	[66]
SPE- CZE-UV	honey	LOD: 23.9–49.3 (μg/kg)	[67]
		**Sulfonamides**	
SPE-CEC-MS	meat	LOD: 0.01–0.14 (μg/L)	[71]
microchip-CE-LIF	milk, meat	LOQ: 0.6–7.7 (μg/L)	[72]
SPE-CZE-ECL	milk, meat	LOD: 0.62–3.14 (μg/mL)	[73]
CZE-UV and CZE-MS	meat, water	LOD: 0.33–180 (μg/L)	[68]
CITP-CZE-UV	beverages, water	LOD: 2.29 (ng/mL)	[69]
	**Aminoglycosides**		
MISPE-FASS-CZE-MS/MS	honey	LOQ: 1.4–94.8 (μg/kg)	[75]
microchip-CE-CCD	standard solutions	LODs: 0.89–3.1 (μg/mL)	[77]
		**Macrolides**	
FASS-MEKC-UV	milk	LOD: 0.002–0.004 (mg/kg)	[83]
R-USAEME-CZE-DAD	chicken fat	LOQ: 22.1–47.0 (μg/kg)	[84]
	**β-lactam antibiotics (penicillins)**		
PPT/CZE-UV	milk	LOQ: 0.04–1.7 (μg/mL)	[89]
ASEI-CEC-MS	milk	LOD: 0.05–0.2 (μg/L)	[90]
	**β-lactam antibiotics (cephalosporins)**		
CZE-UV	complex matrices	LOQ: 4.33–8.00 (μg/mL)	[88]
	**Estrogens**		
SPE-p-CEC-AD	bovine milk, diary products	LOD: 2–50 (ng/mL)	[98]
DLLME-MEKC-ESI-MS/MS	bovine and goat milk, diary products	LOD: 1–61 (μg/L)	[32]
DLLME-MEKC-UV	water	LOD: 0.3–0.6 (μg/L)	[33]
		**NSAIDs**	
DLLME-FASS-CZE-UV	bovine milk, dairy products	LOQ: 10–43.7 (μg/kg)	[99]
SPME-CZE-UV	water	LOQ: 2.91–3.86 (μg/L)	[102]
	**β-agonists**		
SPE-NACE-MS	meat	LOD: 0.3 (ppb)	[103]
CSEI-sweep-MEKC-UV	meat	LOD: 3–5 (ng/g)	[104]
FASI-sweep-MEKC-UV	commercial animal feeds	LOD: 5–20 (ng/mL)	[105]
FESI-CE-C^4^D	pig feed	LOD: 0.02 (mg/L)	[106]

## References

[1] Raza N., Kim K.-H. (2018). Quantification techniques for important environmental contaminants in milk and dairy products. TrAC Trends Anal. Chem..

[2] Commission Regulation (EU) (2010). On pharmacologically active substances and their classification regarding maximum residue limits in foodstuffs of animal origin. Off. J. Eur. Commun..

[B3-molecules-24-04617] 3.U.S. Food and Drug Administration. Chapter I: Food and Drugs Administration, Department of Health and Human Services, Subchapter E: Animal Drugs, Feed, and Related Products, Part 556: Tolerances for residues of New Animal Drugs in food, 2019.

[B4-molecules-24-04617] 4.Codex Alimentarius. Maximum residue limits (MRLs) and risk management recommendations (RMRs) for residues of veterinary drugs in foods, CX/MRL 2-2018.

[5] Lehotay S.J., Chen Y. (2018). Hits and misses in research trends to monitor contaminants in foods. Anal. Bioanal. Chem..

[6] Commission Regulation (EU) (2002). Concerning the performance of analytical methods and interpretation of results. Off. J. Eur. Commun..

[7] Rodriguez E., Moreno-Bondi M.C., Marazuela M.D. (2011). Multiresidue determination of fluoroquinolone antimicrobials in baby foods by liquid chromatography. Food Chem..

[8] Robert C., Brasseur P.-Y., Dubois M., Delahaut P., Gillard N. (2016). Development and validation of rapid multiresidue and multi-class analysis for antibiotics and anthelmintics in feed by ultra-high-performance liquid chromatography coupled to tandem mass spectrometry. Food Addit. Contam.- Part A Chem. Anal. Control Expo. Risk Assess..

[9] Berendsen B.J.A., Meijer T., Mol H.G.J., van Ginkel L., Nielen M.W.F. (2017). A global inter-laboratory study to assess acquisition modes for multi-compound confirmatory analysis of veterinary drugs using liquid chromatography coupled to triple quadrupole, time of flight and orbitrap mass spectrometry. Anal. Chim. Acta.

[10] Mainero Rocca L. (2017). Veterinary drugs residues: A review of the latest analytical research on sample preparation and LC-MS based methods. Food Addit. Contam. Part A Chem. Anal. Control Expo. Risk Assess..

[11] Marazuela M.D., Fanali S., Haddad P.R., Poole C., Riekkola M.-L. (2017). Determination of veterinary drug residues in foods by liquid chromatography-mass spectrometry: Basic and cutting-edge applications. Liquid Chromatography: Applications.

[12] Baglai A. (2018). Enhancing detectability of anabolic-steroid residues in bovine urine by actively modulated online comprehensive two-dimensional liquid chromatography – high-resolution mass spectrometry. Anal. Chim. Acta.

[13] Hernández-Mesa M., Escourrou A., Monteau F., Le Bizec B., Dervilly-Pinel G. (2017). Current applications and perspectives of ion mobility spectrometry to answer chemical food safety issues. TrAC Trends Anal. Chem..

[14] Piňero M.-Y., Bauza R., Arce L. (2011). Thirty years of capillary electrophoresis in food analysis laboratories: Potential applications. Electrophoresis.

[15] Álvarez G., Montero L., Llorens L., Castro-Puyana M., Cifuentes A. (2018). Recent advances in the application of capillary electromigration methods for food analysis and Foodomics. Electrophoresis.

[16] Papetti A., Colombo R., Zhong J., Wang X. (2019). High-performance capillary electrophoresis for food quality evaluation. Evaluation Technologies for Food Quality.

[17] Stavrou I.J., Agathokleous E.A., Kapnissi-Christodoulou C.P. (2017). Chiral selectors in CE: Recent development and applications (mid-2014 to mid-2016). Electrophoresis.

[18] Iacob B.C., Bodoki E., Oprean R. (2014). Recent advances in capillary electrochromatography using molecularly imprinted polymers. Electrophoresis.

[19] Tarongoy F.M., Haddad P.R., Quirino J.P. (2018). Recent developments in open tubular capillary electrochromatography from 2016 to 2017. Electrophoresis.

[20] Týčová A., Ledvina V., Klepárník K. (2017). Recent advances in CE-MS coupling: Instrumentation, methodology, and applications. Electrophoresis.

[21] Klepárník K. (2015). Recent advances in combination of capillary electrophoresis with mass spectrometry: methodology and theory. Electrophoresis.

[22] Elbashir A., Schmitz O.J., Aboul-Enein H.Y. (2017). Application of capillary electrophoresis with capacitively coupled contactless conductivity detection (CE-C4D): An update. Biomed. Chromatogr..

[23] Long C., Deng B., Sun S., Meng S. (2017). Simultaneous determination of chlortetracycline, ampicillin and sarafloxacin in milk using capillary electrophoresis with electrochemiluminescence detection. Food Addit. Contam. Part A Chem. Anal. Control Expo. Risk Assess..

[24] Ferey L., Delaunay N. (2016). Food Analysis on Electrophoretic Microchips. Sep. Purif. Rev..

[25] Breadmore M.C., Wuethrich A., Li F., Phung S.C., Kalsoom U., Cabot J.M., Tehranirokh M., Shallan A.I., Abdul Keyon A.S., See H.H. (2017). Recent advances in enhancing the sensitivity of electrophoresis and electrochromatography in capillaries and microchips (2014–2016). Electrophoresis.

[26] Zhang K., Gan N., Shen Z., Cao J., Hu F., Li T. (2019). Microchip electrophoresis based aptasensor for multiplexed detection of antibiotics in foods via a stir-bar assisted multi-arm junctions recycling for signal amplification. Biosens. Bioelectron..

[27] Šlampová A., Malá Z., Gebauer P.E., Boček P. (2017). Recent progress of sample stacking in capillary electrophoresis (2014–2016). Electrophoresis.

[28] Springer V., Jacksén J., Ek P., Lista A.G., Emmer A. (2014). Determination of fluoroquinolones in bovine milk samples using a pipette-tip SPE step based on multiwalled carbon nanotubes prior to CE separation. J. Sep. Sci..

[29] Wang H., Liu Y., Wei S., Yao S., Zhang J., Huang H. (2016). Selective extraction and determination of fluoroquinolones in bovine milk samples with montmorillonite magnetic molecularly imprinted polymer and capillary electrophoresis. Anal. Bioanal. Chem..

[30] Ramautar R., Somsen G.W., de Jong G.J. (2016). Developments in coupled solid-phase extraction–capillary electrophoresis 2013–2015. Electrophoresis.

[31] Pérez-Rodríguez M., Pellerano R.G., Pezza L., Redigolo Pezza H. (2018). An overview of the main foodstuff sample preparation technologies for tetracycline residue determination. Talanta.

[32] D’Orazio G., Asensio-Ramos M., Hernández-Borges J., Rodríguez-Delgado M.Á., Fanali S. (2015). Evaluation of the combination of a dispersive liquid-liquid microextraction method with micellar electrokinetic chromatography coupled to mass spectrometry for the determination of estrogenic compounds in milk and yogurt. Electrophoresis.

[33] Liu J., Lu W., Liu H., Wu X., Li J., Chen L. (2016). Dispersive liquid-liquid microextraction for four phenolic environmental estrogens in water samples followed by determination using capillary electrophoresis. Electrophoresis.

[34] Kitagawa F., Otsuka K. (2014). Recent applications of on-line sample preconcentration techniques in capillary electrophoresis. J. Chromatogr. A.

[35] Tadeo J.L., Sánchez-Brunete C., González L., Tadeo J.L. (2008). Pesticides: classification and properties. Analysis of Pesticides in Food and Environmental Samples.

[B36-molecules-24-04617] 36.U.S. Food and Drug Administration. Chapter I: Food and Drugs Administration, Department of Health and Human Services, Subchapter E: Animal Drugs, Feed, and Related Products, Part 530: Extralabel drugs use in animals, 2019.

[37] Mudry M.D., Martinez R.A., Nieves M., Carballo M.A. (2011). Biomarkers of geno- toxicity and genomicin stability in a non-human primate, Cebus libidinosus (Cebidae, Platyrrhini), exposed to nitroimidazole derivatives. Mutat. Res. Genet. Toxicol. Environ..

[38] Hernández-Mesa M., Lara F.J., Cruces-Blanco C., García-Campaña A.M. (2015). Determination of 5-nitroimidazole residues in milk by capillary electrochromatography with packed C18 silica beds. Talanta.

[39] Sun H., Wang F., Ai L. (2007). Simultaneous determination of seven nitroimidazole residues in meat byusing HPLC-UV detection with solid-phase extraction. J. Chromatogr. B.

[40] Lin Y., Su Y., Liao X., Yang N., Yang X., Choi M.M.F. (2012). Determination of five nitroimidazole residues in artificial porcine muscle tissue samples by capillary electrophoresis. Talanta.

[41] Tejada-Casado C., Hernández-Mesa M., del Olmo-Iruela M., García-Campaña A.M. (2016). Capillary electrochromatography coupled with dispersive liquid-liquid microextraction for the analysis of benzimidazole residues in water samples. Talanta.

[42] Hashemi B., Zohrabi P., Kim K.-H., Shamsipur M., Deep A., Hong J. (2017). Recent advances in liquid-phase microextraction techniques for the analysis of environmental pollutants. TrAC Trends Anal. Chem..

[43] Hernández-Mesa M., Airado-Rodríguez D., Cruces-Blanco C., García-Campaña A.M. (2014). Novel cation selective exhaustive injection-sweeping procedure for 5-nitroimidazole determination in waters by micellar electrokinetic chromatography using dispersive liquid-liquid microextraction. J. Chromatogr. A.

[44] Tejada-Casado C., Moreno-González D., Lara F.J., García-Campana A.M. (2017). Monsalud del Olmo-Iruela, Determination of benzimidazoles in meat samples by capillary zoneelectrophoresis tandem mass spectrometry following dispersiveliquid–liquid microextraction. J. Chromatogr. A.

[45] Airado-Rodríguez D., Hernández-Mesa M., García-Campaña A.M., Cruces-Blanco C. (2016). Evaluation of the combination of micellar electrokinetic capillary chromatography with sweeping and cation selective exhaustive injection for the determination of 5-nitroimidazoles in egg samples. Food Chem..

[46] Riaz L., Mahmood T., Khalid A., Rashid A., Ahmed Siddique M.B., Kamal A., Coyne M.S. (2018). Fluoroquinolones (FQs) in the environment: A review on their abundance, sorption and toxicity in soil. Chemosphere.

[47] Golomb B.A., Koslik H.J., Redd A.J. (2015). Fluoroquinolone-induced serious, persistent, multisymptom adverse effects. BMJ Case Rep.

[48] Wall G.C., Taylor M.J., Smith H.L. (2018). Prevalence and characteristics of hospital inpatients with reported fuoroquinolone allergy. Int. J. Clin. Pharm..

[49] Czyrski A. (2017). Analytical Methods for Determining Third and Fourth Generation Fluoroquinolones: A Review. Chromatographia.

[50] Piňero M.-Y., Garrido-Delgado R., Bauza R., Arce L., Valcárcel M. (2012). Easy sample treatment for the determination of enrofloxacin and ciprofloxacin residues in raw bovine milk by capillary electrophoresis. Electrophoresis.

[51] De Quirós A.R.-B., Sendón R., Queen T. (2017). Molecularly imprinted polymers: Applications in food science. Molecularly Imprinted Polymers (MIPs): Challenges, Uses and Prospects.

[52] Lombardo-Agüí M., García-Campaña A.M., Gámiz-Gracia L., Cruces Blanco C. (2010). Laser induced fluorescence coupled to capillary electrophoresis for the determination of fluoroquinolones in foods of animal origin using molecularly imprinted polymers. J. Chromatogr. A.

[53] Moreno-González D., Lara F.J., Gámiz-Gracia L., García-Campaña A.M. (2014). Molecularly imprinted polymer as in-line concentrator in capillary electrophoresis coupled with mass spectrometry for the determination of quinolones in bovine milk samples. J. Chromatogr. A.

[54] Lara F.J., García-Capaña A.M., Alés-Barrero F., Bosque-Sendra J.M. (2008). In-line solid-phase extraction preconcentration in capillary electrophoresis-tandem mass spectrometry for the multiresidue detection íof quinolones in meat by pressurized liquid extraction. Electrophoresis.

[55] Xu X., Liu L., Jia Z., Shu Y. (2015). Determination of enrofloxacin and ciprofloxacin in foods of animal origin by capillary electrophoresis with field amplified sample stacking–sweeping technique. Food Chem..

[56] Deng Y., Gasilova N., Qiao L., Zhou Y.-L., Zhang X.-X., Girault H.H. (2014). Highly sensitive detection of five typical fluoroquinolones in low-fat milk byfield-enhanced sample injection-based CE in bubble cell capillary. Electrophoresis.

[57] Herrera-Herrera A.V., Hernández-Borges J., Borges-Miquel T.M., Rodríguez-Delgado M.A. (2010). Dispersive liquid-liquid microextraction combined with nonaqueous capillary electrophoresis for the determination of fluoroquinolone antibiotics in waters. Electrophoresis.

[58] Springer V.H., Lista A.G. (2015). In-line coupled single drop liquid–liquid–liquid microextraction with capillary electrophoresis for determining fluoroquinolones in water samples. Electrophoresis.

[59] Ma T.Y., Vickroy T.W., Shien J.H., Chou C.C. (2012). Improved nonaqueous capillary electrophoresis for tetracyclines at subparts per billion level. Electrophoresis.

[60] Deng B., Xu Q., Lu H., Ye L., Wang Y. (2012). Pharmacokinetics and residues of tetracycline in crucian carp muscle using capillary electrophoresis on-line coupled with electrochemiluminescence detection. Food Chem..

[61] Díaz-Quiroz C.A., Hernández-Chávez J.F., Ulloa-Mercado G., Gortáres-Moroyoqui P., Martínez-Macías R., Meza-Escalante E., Serrano-Palacios D. (2018). Simultaneous quantification of antibiotics in wastewater from pig farms by capillary electrophoresis. J. Chromatogr. B.

[62] Islas G., Rodriguez J.A., Perez-Silva I., Miranda J.M., Ibarra I.S. (2018). Solid-Phase Extraction and Large-Volume Sample Stacking-Capillary Electrophoresis for Determination of Tetracycline Residues in Milk. J. Anal. Methods Chem..

[63] Moreno-González D., Lupión-Enríquez I., Garcia-Campaňa A.M. (2016). Trace determination of tetracyclines in water samples by capillary zone electrophoresis combining off-line and on-line sample preconcentratio. Electrophoresis.

[64] Wu X., Xu Z., Huang Z., Shao C. (2016). Large volume sample stacking of cationic tetracycline antibiotics toward 10 ppb level analysis by capillary electrophoresis with UV detection. Electrophoresis.

[65] Mu G., Liu H., Xu L., Tian L., Luan F. (2012). Matrix Solid-Phase Dispersion Extraction and Capillary Electrophoresis Determination of Tetracycline Residues in Milk. Food Anal. Methods.

[66] Zhou C., Deng J., Shi G., Zhou T. (2017). β-cyclodextrin-ionic liquid polymer based dynamically coating for simultaneous determination of tetracyclines by capillary electrophoresis. Electrophoresis.

[67] Casado-Terrones S., Segura-Carretero A., Busi S., Dinelli G., Fernández-Gutiérrez A. (2007). Determination of tetracycline residues in honey by CZE with ultraviolet absorbance detection. Electrophoresis.

[68] Amin N.C., Blanchin M.D., Aké M., Fabre H. (2013). Capillary electrophoresis methods for the analysis of antimalarials. Part II. Achiral separative methods. J. Chromatogr. A.

[69] Mikus P., Maráková K., Veizerová L., Piešt’anský J. (2011). Determination of quinine in beverages by online coupling capillary isotachophoresis to capillary zone electrophoresis with UV spectrophotometric detection. J. Sep. Sci..

[70] Kluska M., Marciniuk-Kluska A., Prukala D., Prukala W. (2016). Analytics of quinine and its derivatives. Crit. Rev. Anal. Chem..

[71] Cheng Y.J., Huang S.H., Singco B., Huang H.-Y. (2011). Analyses of sulfonamide antibiotics in meat samples by on-line concentration capillary electrochromatography-mass spectrometry. J. Chromatogr. A.

[72] Wang L., Wu J., Wang Q., He C., Zhou L., Wang J., Pu Q. (2012). Rapid and sensitive determination of sulfonamide residues in milk and chicken muscle by microfluidic chip electrophoresis. J. Agric. Food Chem..

[73] Dai T., Duan J., Li X., Xu X., Shi H., Kang W. (2017). Determination of Sulfonamide Residues in Food by Capillary Zone Electrophoresis with On-Line Chemiluminescence Detection Based on an Ag(III) Complex. Int. J. Mol. Sci..

[74] Farouk F., Azzazy H.M.E., Niessen W.M.A. (2015). Challenges in the determination of aminoglycoside antibiotics, a review. Anal. Chim. Acta.

[75] Moreno-González D., Lara F.J., Jurgovská N., Gámiz-Gracia L., García-Campana A.M. (2015). Determination of aminoglycosides in honey by capillary electrophoresis tandem mass spectrometry and extraction with molecularly imprinted polymers. Anal. Chim. Acta.

[76] Liu H., Li N., Liu X., Qian Y., Qiu J., Wang X. (2019). Poly(N-acryloyl-glucosamine-co-methylenebisacrylamide)-based hydrophilic magnetic nanoparticles for the extraction of aminoglycosides in meat samples. J. Chromatogr. A.

[77] Zhu G., Bao C., Liu W., Yan X., Liu L., Xiao J., Chen C. (2019). Rapid detection of ags using microchip capillary electrophoresis contactless conductivity detection. Curr. Pharm. Anal..

[78] Commission Regulation (EU) (1996). On measures to monitor certain substances and residues thereof in live animals and animal products and repealing Directives 85/358/EEC and 86/469/EEC and Decisions 89/187/EEC and 91/664/EEC. Off. J. Eur. Commun..

[79] Dinos G.P. (2017). The macrolide antibiotic renaissance. Br. J. Pharmacol..

[80] Pyörälä S., Baptiste K.E., Catry B., van Duijkeren E., Greko C., Moreno M.A., Pomba M.C., Rantala M., Ružauskas M., Sanders P. (2014). Macrolides and lincosamides in cattle and pigs: Use and development of antimicrobial resistance. Vet. J..

[81] Şanli S., Palabiyik I.M., Şanli N., Guzel-Seydim Z.B., Alsancak G. (2011). Optimization of the experimental conditions for macrolide antibiotics in high performance liquid chromatography by using response surface methodology and determination of tylosin in milk samples. J. Anal. Chem..

[82] Dickson L.C., O’Byrne C., Chan W. (2012). A quantitative method for residues of macrolide antibiotics in porcine kidney by liquid chromatography/ tandem mass spectrometry. J. AOAC Int..

[83] Hong Y.-Q., Guo X., Chen G.-H., Zhou J.-W., Zou X.-M., Liao X., Hou T. (2018). Determination of five macrolide antibiotic residues in milk by micellar electrokinetic capillary chromatography with field amplified sample stacking. J. Food Saf..

[84] Lorenzetti A.S., Lista A.G., Domini C.E. (2019). Reverse ultrasound-assisted emulsification-microextraction of macrolides from chicken fat followed by electrophoretic determination. LWT-Food Sci. Technol..

[85] Cameron-Veas K., Solà-Ginés M., Moreno M.A., Fraile L., Migura-Garcia L. (2015). Impact of the use of β-lactam antimicrobials on the emergence of Escherichia coli isolates resistant to cephalosporins under standard pig-rearing conditions. Appl. Environ. Microbiol..

[86] Cameron-Veas K., Moreno M.A., Fraile L., Migura-Garcia L. (2016). Shedding of cephalosporin resistant Escherichia coli in pigs from conventional farms after early treatment with antimicrobials. Vet. J..

[87] Piñero M.-Y., Bauza R., Arce L., Valcárcel M. (2014). Determination of penicillins in milk of animal origin by capillary electrophoresis: Is sample treatment the bottleneck for routine laboratories. Talanta.

[88] Hancu G., Sasebeşi A., Rusu A., Kelemen H., Ciurba A. (2015). Study of the electrophoretic behavior of cephalosporins by Capillary Zone Electrophoresis. Adv. Pharm. Bull..

[89] Li M.H., He W., Zhang L., Duan C. (2015). Analysis of penicillin and its β-lactamase hydrolysis products in milk using capillary zone electrophoresis. Anal. Methods.

[90] Liu W.-L., Wu C.-Y., Li Y.-T., Huang H.-Y. (2012). Penicillin analyses by capillary electrochromatography-mass spectrometry with different charged poly(stearylmethacrylate–divinylbenzene) monoliths as stationary phases. Talanta.

[91] Tong J., Rao Q., Zhu K., Jang Z., Ding S. (2009). Simultaneous determination of five tetracycline and macrolide antiobiotics in feeds using HPCE. J. Sep. Sci..

[B92-molecules-24-04617] 92.Regulation (EC) No 1831/2003, Annex I: list of additives, Edition 11/2019 (277).

[93] Vera-Candioti L., Olivieri A.C., Goicoechea H.C. (2010). Development of a novel strategy for preconcentration of antibiotic residues in milk and their quantitation by capillary electrophoresis. Talanta.

[94] Kowalski P., Plenis A., Oledzka I., Konieczna L. (2011). Optimization and validation of the micellar electrokinetic capillary chromatographic method for simultaneous determination of sulfonamide and amphenicol-type drugs in poultry tissue. J. Pharm. Biomed. Anal..

[95] Liu L., Wan Q., Xu X., Duan S., Yang C. (2017). Combination of micelle collapse and field-amplified sample stacking in capillary electrophoresis for determination of trimethoprim and sulfamethoxazole in animal-originated foodstuffs. Food Chem..

[96] He T., Xu Z., Ren J. (2019). Pressure-assisted electrokinetic injection stacking for seven typical antibiotics in waters to achieve μg/L level analysis by capillary electrophoresis with UV detection. Microchem. J..

[97] Adeel M., Song X., Wang Y., Francis D., Yang Y. (2017). Environmental impact of estrogens on human, animal and plant life: A critical review. Environ. Int..

[98] Wu W., Yuan X., Wu X., Lin X., Xie Z. (2010). Analysis of phenolic xenoestrogens by pressurized CEC with amperometric detection. Electrophoresis.

[99] European Commission (2004). Council Regulation 324/2004/EC.2005. Off. J. Eur. Commun..

[100] Alshana U., Göğer N.G., Ertaş N. (2013). Dispersive liquid–liquid microextraction combined with field-amplified sample stacking in capillary electrophoresis for the determination of non-steroidal anti-inflammatory drugs in milk and dairy products. Food Chem..

[101] Shishov A., Nechaeva D., Bulatov A. (2019). HPLC-MS/MS determination of non-steroidal anti-inflammatory drugs in bovine milk based on simultaneous deep eutectic solvents formation and its solidification. Microchem. J..

[102] Espina-Benitez M., Araujo L., Prieto A., Navalón A., Vílchez J.L., Valera P., Zambrano A., Dugas V. (2017). Development of a new microextraction fiber combined to on-line sample stacking capillary electrophoresis UV detection for acidic drugs determination in real water samples. Int. J. Environ. Res. Public Health.

[103] Anurukvorakun O., Buchberger W., Himmelsbach M., Klampel C.W., Suntornsuk L. (2010). A sensitive non-aqueous capillary electrophoresis-mass spectrometric method for multiresidue analyses of beta-agonists in pork. Biomed. Chromatogr..

[104] Wang C.-C., Lu C.-C., Chen Y.-L., Cheng H.-L., Wu S.-M. (2013). Chemometric optimization of cation-selective exhaustive injection sweeping micellar electrokinetic chromatography for quantification of ractopamine in porcine meat. J. Agric. Food Chem..

[105] Hsieh S.-Y., Wang C.-C., Kou H.-S., Wu S.M. (2017). Dialkyl anionic surfactant in field-amplified sample injection andsweeping-micellar electrokinetic chromatography for determinationof eight leanness-promoting β-agonists in animal feeds. J. Pharm. Biomed. Anal..

[106] Gao F., Wu M., Zhang Y., Wang G., Wang Q., He P., Fang Y. (2014). Sensitive determination of four β2-agonists in pig feed by capillary electrophoresis using on-line sample preconcentration with contactless conductivity detection. J. Chromatogr. B.

